# Retinoids in scarless skin regeneration: from molecular mechanisms to therapeutic strategies

**DOI:** 10.3389/fcell.2025.1683851

**Published:** 2025-09-29

**Authors:** Kang Wang, Ziting Yang, Yunqi Ma, Wenhui Liu, Guangshuai Li, Xuewen Xu, Qingfeng Li

**Affiliations:** ^1^ Department of Plastic and Burn Surgery, West China Hospital, West China School of Medicine, Sichuan University, Chengdu, China; ^2^ Department of Plastic and Reconstructive Surgery, Shanghai Ninth People’s Hospital, Shanghai Jiao Tong University School of Medicine, Shanghai, China; ^3^ Department of Plastic and Reconstructive Surgery, The First Affiliated Hospital of Zhengzhou University, Zhengzhou, China; ^4^ Technology and Media University of Henan Kaifeng, Kaifeng, China

**Keywords:** retinoids, fibroblasts, skin regeneration, scarless wound healing, hair follicle neogenesis, regenerative biomaterials

## Abstract

Scarless skin regeneration remains one of the most ambitious goals in regenerative medicine. Unlike fibrotic healing, which results in excessive collagen accumulation and functional impairment, true regeneration restores both the structural integrity and physiological function of skin, including the reconstitution of hair follicles and other appendages. Retinoids, a broad class of natural and synthetic vitamin A derivatives, have attracted increasing attention for their potential to modulate wound repair at multiple levels. These compounds regulate a diverse array of biological processes, including epidermal differentiation, fibroblast activation, immune response, and extracellular matrix remodeling. This review provides a comprehensive overview of how retinoids coordinate cellular and molecular events across key skin compartments during healing. Retinoids have been reported to suppress TGF-β1/Smad signaling, inhibit myofibroblast differentiation, and restore matrix homeostasis, thereby exerting anti-fibrotic effects. In addition, retinoid-based therapies enhance re-epithelialization, stimulate angiogenesis, and promote dermal regeneration when incorporated into advanced biomaterial systems. Recent studies further demonstrate that retinoids can support skin appendage regeneration, including *de novo* hair follicle formation, a hallmark of functional repair typically absent in adult wounds. In view of converging evidence from developmental biology, stem cell research, and regenerative engineering, retinoids present a promising pharmacological strategy in reduced-scarring healing and functional skin regeneration.

## 1 Introduction

**FIGURE 1:**
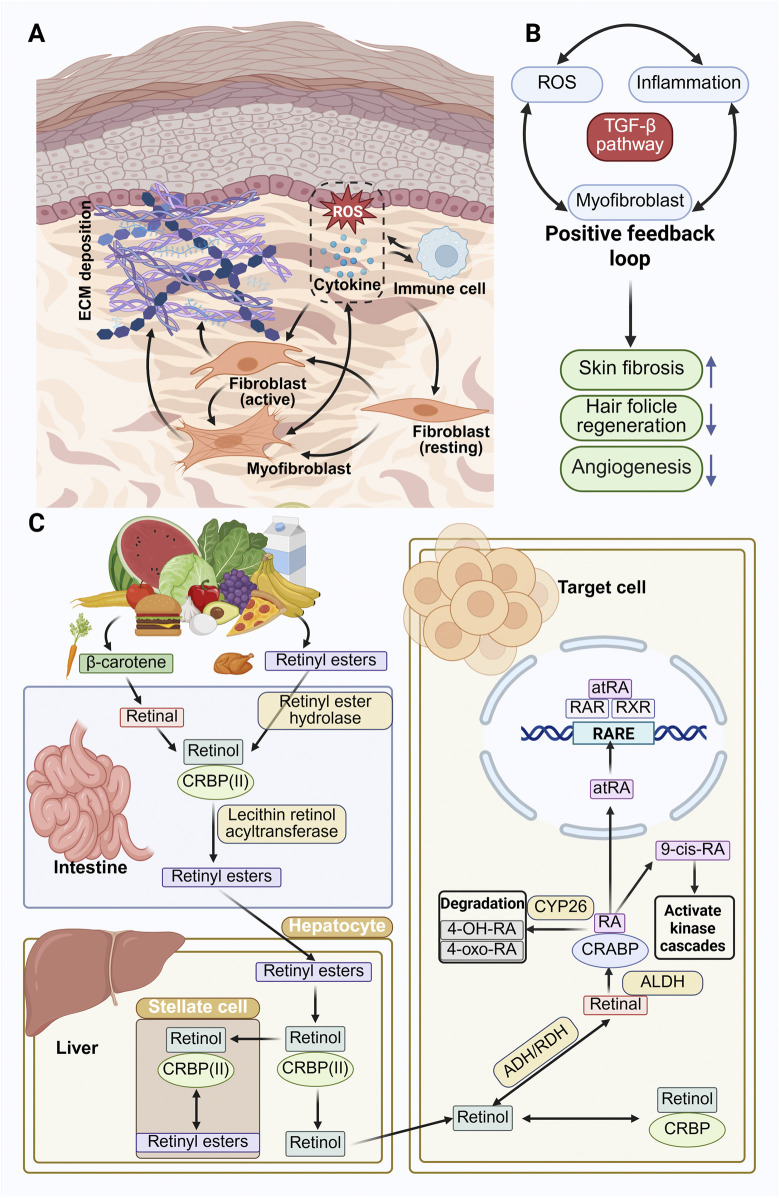
Overview of retinoic acid metabolism and skin fibrosis mechanism **(A)** Schematic illustration of fibrotic wound healing. Following injury, infiltrating immune cells release cytokines and reactive oxygen species (ROS), which amplify local inflammation and tissue stress. Resident dermal fibroblasts are activated and differentiate into myofibroblasts under the influence of ROS and transforming growth factor-beta 1 (TGF-β1). These myofibroblasts migrate into the wound site, deposit excessive extracellular matrix (ECM), and secrete profibrotic mediators, leading to collagen accumulation, matrix stiffening, and scar formation. **(B)** Positive feedback loop driving fibrosis. Crosstalk among ROS, inflammation, TGF-β1 signaling, and myofibroblast activation establishes a self-perpetuating cycle that reinforces ECM overproduction. This process promotes skin fibrosis while impairing regenerative events such as angiogenesis and hair follicle neogenesis. **(C)** Metabolism and Signaling of Retinoids: The uptake and metabolism of retinoids can be broadly divided into three stages based on organ localization. In the first stage, within the gastrointestinal tract, β-carotene and retinyl esters are absorbed and converted into retinol. Retinol then binds to cellular retinol-binding protein II (CRBPII) to prevent premature oxidation. The retinol–CRBPII complex is subsequently re-esterified into retinyl esters, packaged into chylomicrons, and transported through the lymphatic system into systemic circulation. In the second stage, hepatocytes uptake circulating retinyl esters from the bloodstream. These are hydrolyzed back to retinol, a portion of which is stored in hepatic stellate cells as retinyl esters within cytoplasmic lipid droplets, while another portion is secreted back into plasma. In the third stage, plasma retinol is taken up by target cells. Once inside, it may bind to cellular retinol-binding proteins (CRBPs) or undergo sequential oxidation by alcohol dehydrogenases and retinol dehydrogenases (ADH/RDH) to form retinaldehyde (retinal). Retinal is then irreversibly oxidized by aldehyde dehydrogenases (ALDHs) to form RA, including atRA, 9-cis-RA, and 13-cis-RA. atRA binds to CRABPs and translocates to the nucleus, where it interacts with nuclear receptors RAR and RXR to regulate gene transcription. In parallel, 9-cis-RA can activate intracellular kinase cascades. Retinoic acids are further metabolized by cytochrome P450 enzymes (particularly CYP26 family) into inactive oxidative metabolites such as 4-hydroxyretinoic acid (4-OH-RA) and 4-oxoretinoic acid (4-oxo-RA).

The skin is the largest organ of the human body, functioning as a barrier against environmental insults while maintaining immune surveillance, thermoregulation, and sensory integration (Joshi et al., 2025; Lopez-Ojeda et al., 2022). It is composed of three primary layers (epidermis, dermis, and hypodermis) each containing distinct cellular components that coordinate structural support, immune defense, and regenerative capacity (Figure 1A). The epidermis is populated primarily by keratinocytes and houses epidermal stem cells within the basal layer. The underlying dermis contains fibroblasts, endothelial cells, and a dynamic ECM, while the skin appendages such as hair follicles, sebaceous glands, and sweat glands originate at the epidermal-dermal interface. These specialized compartments play critical roles in maintaining skin homeostasis and facilitating wound healing.

Upon injury, adult skin typically undergoes fibrotic repair, restoring the epidermal barrier but often resulting in permanent scar formation. Such scarring can compromise tissue function, mechanical strength, and aesthetic appearance (Peña and Martin, 2024). During fibrotic healing, immune cells infiltrate the wound bed and release large amounts of cytokines, triggering a sustained inflammatory response. In parallel, local levels of reactive oxygen species (ROS) rise rapidly, further amplifying tissue stress. Resident dermal fibroblasts are activated by profibrotic mediators, particularly transforming growth factor-beta 1 (TGF-β1) and ROS, and subsequently differentiate into myofibroblasts (Figures 1A,B). These myofibroblasts migrate into the wound site, where they deposit excessive extracellular matrix (ECM) and perpetuate a positive feedback loop (Figure 1B). This cycle not only drives fibrotic remodeling but also impairs key regenerative processes such as angiogenesis and hair follicle neogenesis (Wang et al., 2023). Despite advances in wound care, current interventions rarely enable full-thickness tissue regeneration.

Scarless skin healing, the aspirational goal of regenerative dermatology, is defined by two key hallmarks: the suppression of fibrotic remodeling and the regeneration of skin appendages, particularly hair follicles (Li et al., 2024). These features are essential not only for restoring skin integrity but also for reinstating its full physiological function. Retinoids, which include both natural and synthetic derivatives of vitamin A, have already been widely applied in cosmeceuticals and are well recognized for their anti-aging efficacy (Siddiqui et al., 2024; Böhm et al., 2025). More recently, they have emerged as compelling candidates in the field of regenerative dermatology. The bioactive form of vitamin A, retinoic acid (RA), is synthesized endogenously from dietary precursors such as retinyl esters and β-carotene (Bohn et al., 2023). RA governs a broad range of biological processes including epithelial differentiation, embryonic patterning, immune modulation, and ECM remodeling (Wang et al., 2020; Szymański et al., 2020). Through regulating these biological events, retinoids display unique function that concurrently target the two key hallmarks of scarless healing: inhibiting fibrogenesis and promoting skin appendage regeneration (Wang et al., 2020; Wen et al., 2025). This review examines the therapeutic potential of retinoids in promoting scarless skin regeneration by targeting both fibrosis suppression and appendage renewal, with a focus on their molecular mechanisms and translational prospects.

## 2 Biological basis of retinoids in skin

### 2.1 Synthesis, metabolism, and signaling of RA

As shown in Figure 1C, RA is the primary bioactive metabolite of vitamin A (Osanai et al., 2023). Although RA itself is not directly obtained from the diet, it is synthesized intracellularly through a multistep enzymatic process (Gudas, 2022a). Various dietary forms of vitamin A, including retinyl esters and provitamin A carotenoids such as β-carotene, can serve as precursors (Gudas, 2022a). These compounds are first hydrolyzed to retinol (Lavudi et al., 2023). The final and rate-limiting step is the irreversible oxidation of retinaldehyde to all-trans retinoic acid (atRA), catalyzed by retinaldehyde dehydrogenases RALDH1, RALDH2, and RALDH3, encoded by the ALDH1A1–3 genes (Thompson et al., 2019). RA exerts its biological effects by binding to RA receptors (RARα, RARβ, RARγ), which form heterodimers with retinoid X receptors (RXRs). These complexes interact with RA response elements (RAREs) in the promoter regions of target genes to regulate transcription (Lavudi et al., 2023; Cunningham and Duester, 2015). Intracellularly, RA is further regulated by cellular RA-binding proteins (CRABPs). In addition, cytochrome P450 enzymes, such as CYP26A1 and CYP26B1, metabolize RA into inactive derivatives, providing negative feedback to prevent excessive signaling. This tightly controlled system ensures that RA levels remain within a precise range, enabling context-specific responses to injury and repair signals (Das et al., 2013; Hu et al., 2024).

### 2.2 Biological effects of retinoids across skin compartments

The regenerative effects of RA in skin repair arise from its ability to modulate key cellular populations and signaling pathways across multiple layers of tissue. RA orchestrates healing by regulating epidermal stem cells, dermal fibroblasts, and immune cells.

In the epidermis, RA promotes re-epithelialization by enhancing keratinocyte proliferation and differentiation, effects that have long been established (Saitou et al., 1995; Fuchs and Green, 1981). Both retinol and RA have been shown to increase epidermal thickness and upregulate the expression of collagen types I and III in human skin (Kon et al., 2016). Notably, the fourth-generation retinoid seletinoid G has been demonstrated to stimulate keratinocyte proliferation and migration, thereby accelerating wound re-epithelialization (Lee et al., 2020). In the dermis, RA attenuates fibrotic activation of fibroblasts. It suppresses the expression of key fibrogenic markers such as α-SMA, COL1A1, and COL3A1 through downregulation of the TGF-β1/Smad signaling axis (Lin et al., 2023). In a murine ear wound model, topical application of the RAR agonist tazarotene enhanced wound closure and led to regeneration of skin appendages, including newly formed hair follicles and mature collagen fibers (Al et al., 2016). At the ECM level, RA promotes matrix turnover by upregulating matrix-degrading enzymes such as MMP-3 and MMP-13, while downregulating tissue inhibitors of metalloproteinases (TIMPs), thus slowing fibrotic progression (Kartasheva-Ebertz et al., 2021; Sorg et al., 2006). These effects have been confirmed in animal models, where RA-treated wounds exhibit reduced scar formation and restoration of near-normal tissue architecture. The immune microenvironment is another critical target of RA (Mora et al., 2008). RA has been shown to influence macrophages, T cells, and B cells, and plays an essential role in maintaining immune homeostasis during tissue repair (Oliveira et al., 2018; Erkelens and Mebius, 2017). As reviewed in detail by Oliveira et al. (Oliveira et al., 2018), RA modulates both innate and adaptive immune responses. In photoaged skin, vitamin A derivatives reduce the production of pro-inflammatory cytokines (Riahi et al., 2016). In psoriasis, which is an inflammatory dermatosis characterized by leukocyte infiltration, topical tazarotene cream has demonstrated notable therapeutic efficacy (Weinstein et al., 2003).

## 3 Mechanistic potential of retinoids in scarless skin healing

### 3.1 Anti-fibrotic mechanisms

As shown in Figures 1A,B, fibrotic scarring in skin wounds arises primarily from the pathological activation of dermal fibroblasts into myofibroblasts, sustained stimulation of the TGF-β1/Smad pathway, and excessive accumulation of ECM (Peña and Martin, 2024). These processes ultimately result in disorganized tissue architecture, stiffness, and functional impairment (Martin and Nunan, 2015). RA, the active metabolite of vitamin A, has been shown to interfere with multiple steps in fibrogenesis (Barber et al., 2014; Jumper et al., 2016). Its anti-fibrotic actions, observed across various organs including the lung, liver, and kidney, involve inhibition of TGF-β signaling, suppression of myofibroblast differentiation, and enhancement of ECM degradation (Wang et al., 2020). To be specific, in pulmonary fibrosis models, RA reduces oxidative stress, regulates inflammatory cytokines, and attenuates ECM deposition (Gokey et al., 2021; Eleraky et al., 2021; Lu et al., 2022). In the liver fibrosis, RA suppresses IL-17A production and downregulates IL-6R and IL-23R expression, thereby limiting hepatic stellate cell activation and collagen synthesis (Kartasheva-Ebertz et al., 2021; Xiong et al., 2023; Cassim and Zhang, 2023). Although mechanistic insights into RA’s anti-fibrotic activity in the skin are still emerging, early studies have demonstrated that RA, particularly atRA, inhibits fibroblast proliferation and collagen type I production in human dermal cultures (Daly and Weston, 1986). However, the anti-fibrotic effects of RA are not universally consistent (Zhou et al., 2012). Some studies report context-dependent pro-fibrotic outcomes (Hwang et al., 2021), such as increased collagen synthesis and ECM accumulation under specific concentrations, delivery methods, or cellular states (Zhou et al., 2012; Czuba et al., 2021; Möller-Hackbarth et al., 2021; Rankin et al., 2013; Jalian et al., 2008). These paradoxical findings emphasize the need to clarify RA’s “conditional specificity” in cutaneous fibrosis, which will be essential for safe and effective clinical application.

#### 3.1.1 RA and modulation of TGF-β1/smad signaling

The TGF-β1/Smad signaling pathway is considered the central driver of dermal fibrosis (Wang et al., 2023). Following skin injury, TGF-β1 levels rise sharply, activating Smad2/3 phosphorylation and nuclear translocation. Within the dermis, this cascade triggers the transition of fibroblasts into myofibroblasts and promotes the production of type I and type III collagen, thereby reinforcing the fibrotic microenvironment (Figure 1C) (Wang et al., 2023; Chang et al., 2025). Furthermore, the accumulation of type I collagen in the dermis can activate integrin-mediated signaling, which in turn stimulates the proliferation and differentiation of epidermal keratinocytes, ultimately shaping the characteristic histological architecture of scar tissue (Chang et al., 2025).

RA has been shown to suppress TGF-β1 expression and reduce phosphorylation of Smad1/5/8, thereby interrupting the pathway’s activation loop (Song et al., 2013; Shimono et al., 2011). *In vitro* studies on human fetal palatal mesenchymal cells demonstrated that RA dose-dependently inhibited the synthesis of ECM components, such as fibronectin and tenascin C, through downregulation of MMP2 and TIMP2 mediated by suppression of TGF-β/Smad signaling (Li et al., 2014). Other reports confirm that RXR agonists inhibit Smad nuclear translocation, suppress fibroblast activation, and reduce collagen production in TGF-β1-stimulated fibroblasts (Lin et al., 2023). These findings suggest that RA and RXR-targeted ligands act synergistically to exert anti-fibrotic effects in the dermis by disrupting the TGF-β1 axis. In addition, within the epidermis, retinoids have been shown to play a pivotal role in maintaining homeostasis and promoting regeneration. Supplementation with retinoid metabolites revitalizes epidermal cells, enhancing their structural and functional integrity (Wu et al., 2025; Kim et al., 1992; Quan, 2023). The regulatory effects of retinoic acid on epidermal biology may, at least in part, be mediated through interactions with the TGF-β1 signaling pathway (Kim et al., 1992).

#### 3.1.2 Regulation of myofibroblast differentiation and collagen deposition

Myofibroblast activation is a pivotal event in dermal fibrosis, characterized by α-smooth muscle actin (α-SMA) expression and elevated contractile and collagen-synthetic activity. RA has been reported to inhibit pro-inflammatory fibroblast (PIF) activation and promote differentiation into less fibrogenic mesenchymal fibroblast phenotypes (Xiao et al., 2024). Delivery of RA via nanoparticles has been shown to reduce α-SMA levels and collagen accumulation in fibrotic tissues (Xia et al., 2023). In systemic sclerosis models, atRA reduced the expression of fibrosis markers including Fra2, collagen I, and α-SMA (Pi et al., 2023). Moreover, RA downregulates a broad set of ECM-related genes such as fibronectin-1, thrombospondin-1, tenascin C, integrins, and laminins (Du et al., 2013). Suppression of these components may prevent matrix crosslinking and stiffness, facilitating a tissue environment conducive to regeneration.

Recent work by Correa-Gallegos et al. (2023) revealed that RA gradients within wound beds shape fibroblast fate decisions. In early inflammation, CD201^+^ progenitor fibroblasts upregulate Aldh1a3 and Rdh10, enzymes critical for RA biosynthesis, which in turn activate RARγ and favor the emergence of pro-inflammatory fibroblasts over myofibroblasts. Exogenous RA or CYP26B1 inhibition further suppressed myofibroblast formation, reduced wound contraction, and minimized scar formation. This study provides a compelling mechanistic link between local RA signaling and myofibroblast lineage specification, reinforcing the rationale for RA-based anti-fibrotic therapies.

### 3.2 Skin appendage regeneration

In recent years, RA has emerged as a promising regulator of skin appendage regeneration, especially in the context of wound-induced hair follicle neogenesis (WIHN). As a classical morphogen, RA participates in epithelial-mesenchymal crosstalk and stem cell activation, positioning it as a central signal in the transition from fibrotic repair to structural regeneration (Ankawa and Fuchs, 2022).

#### 3.2.1 Role of RA in WIHN

Hair follicles, sebaceous glands, and sweat glands are essential components of fully functional skin. In adult mammals, these structures rarely regenerate after full-thickness injury, leading to functionally deficient scars. However, WIHN, a phenomenon first characterized in murine dorsal wounds, demonstrates that appendage regeneration is possible under specific conditions (Ito et al., 2007).

RA has been shown to play a critical role in this process (Bhoopalam et al., 2020; Goggans et al., 2024). Endogenous RA synthesis is induced by double-stranded RNA signaling through Toll-like receptor 3 (TLR3), which stimulates RA production and promotes WIHN. Kim et al. showed that RA is essential for WIHN in mice (Kim et al., 2019). In human skin, laser resurfacing similarly activates RA production, suggesting translational relevance (Kim et al., 2019). Standardized WIHN models developed by Garza and colleagues have further validated RA’s involvement in appendage regeneration (Xue et al., 2022). Recent studies indicate that RA can restore hair follicle stem cell (HFSC) identity (Tierney et al., 2024). Through RARγ/RXRα signaling, RA activates lineage-determining factors such as SOX9 and suppresses epidermal markers like KLF5, thereby steering HFSCs back toward a hair follicle fate (Tierney et al., 2024). These findings suggest that RA may serve as a “lineage-resetting” signal in early wound healing, providing a mechanistic window for targeted intervention.

#### 3.2.2 Crosstalk between RA and Wnt/β-catenin, shh, and BMP signaling

RA does not act in isolation but intersects with key developmental pathways, notably Wnt/β-catenin, Sonic hedgehog (Shh), and bone morphogenetic protein (BMP) signaling. Activation of Wnt/β-catenin is essential for HFSC activation and anagen entry. In a recent study on androgenetic alopecia, Wen et al. demonstrated that RA reactivates dormant HFSCs and prolongs hair cycling through Wnt enhancement (Wen et al., 2025). Clinical observations supported RA’s potential in restoring follicular activity in early-stage AGA patients (Wen et al., 2025). In developmental biology, RA has been shown to interact synergistically with multiple signaling pathways, including the Shh axis. RA plays a pivotal role in embryogenesis and the regenerative development of various tissues and organs, as demonstrated in multiple studies (Lukonin et al., 2020; Rekler and Kalcheim, 2022; Niederreither and Dollé, 2008; Wu et al., 2022). The Shh pathway is a key regenerative signal during embryonic hair follicle morphogenesis and WIHN. Through activation of downstream effectors such as Ptch1 and Gli1, Shh signaling induces bidirectional activation of both epidermal and dermal stem cells, thereby initiating the formation of new follicular units (Wier and Garza, 2020; Liu et al., 2022). Similarly, the BMP pathway plays a critical role in cutaneous wound healing, hair follicle cycling, and spatial patterning (Plikus et al., 2017; Hu et al., 2021). These developmental insights offer valuable mechanistic parallels for understanding how RA may coordinate with conserved morphogenetic pathways to promote skin regeneration and appendage restoration.

## 4 Therapeutic applications and translational opportunities

The biological effects of RA are mediated through its interaction with RARs, which are members of the nuclear receptor superfamily of transcription factors (Figure 1C). These receptors include three main isoforms: RARα, RARβ, and RARγ (di Masi et al., 2015). Each of these can form homodimers or heterodimers with retinoid X receptors (RXRα, RXRβ, and RXRγ), enabling gene regulation through RA response elements (RARE) (Di Masi et al., 2015). Non-selective activation of all three RAR isoforms has been linked to adverse cutaneous effects, such as skin irritation, erythema, and desquamation. Among these, RARγ is the most abundantly expressed isoform in the epidermis, accounting for approximately 90 percent of total RAR expression in this layer (Di Masi et al., 2015). RARγ plays a central role in controlling terminal differentiation of keratinocytes. As a result, selective activation of RARγ is considered a key strategy for maximizing therapeutic benefit while minimizing systemic and local side effects.

Retinoids are routinely used to treat acne, photoaging, psoriasis, pigmentary disorders, and certain skin cancers (Chen et al., 2014; Gudas, 2022b; Paichitrojjana and Paichitrojjana, 2023). Currently, retinoids are grouped into four generations based on chemical structure and receptor selectivity. First- and second-generation agents (e.g., tretinoin, isotretinoin, etretinate, and acitretin) bind non-selectively to all RAR subtypes and are associated with systemic toxicity and teratogenicity (Chambon, 1996). Third-generation retinoids (such as adapalene and tazarotene) demonstrate improved receptor selectivity, particularly for RARβ and RARγ, resulting in better tolerability. Trifarotene, a fourth-generation compound, is a highly selective RARγ agonist that offers efficacy in truncal and facial acne with a favorable safety profile due to rapid hepatic metabolism (Gudas, 2022b; Aubert et al., 2018; Wagner et al. 2020).

Table 1 summarizes the applications, dosage considerations, and administration routes of various generations of retinoids in skin diseases. In clinical dermatology, topical adapalene is a first-line therapy for mild acne, while oral isotretinoin is typically prescribed for severe or treatment-resistant cases (Wagner et al. 2020; Kolli et al., 2019). Topical retinoid formulations are also extensively applied in the management of skin aging, where they improve dermal collagen synthesis, reduce fine wrinkles, and counteract photoaging (Kon et al., 2016; Kligman et al., 1986; Talwar et al., 1995). Tretinoin was the first retinoid to receive FDA approval for photoaged skin and has demonstrated significant efficacy in reducing wrinkles, mottled hyperpigmentation, and surface roughness (Mukher et al., 2006; Yoham and Casadesus, 2025). Combination products such as Tri-Luma, which includes fluocinolone acetonide, hydroquinone, and tretinoin, are approved for the treatment of melasma and hyperpigmentation (Torok, 2006; McKesey et al., 2020). In psoriasis, systemic acitretin is often combined with phototherapy to reduce cumulative UV exposure, while topical tazarotene provides local anti-inflammatory effects (Lebwohl et al., 2004; Ogawa et al., 2018; Mehta and Lim, 2016; van de Kerkhof and de Rooij, 1997). Beyond inflammatory conditions, retinoids play a role in skin oncology. Bexarotene, a selective RXR agonist, is approved for cutaneous T-cell lymphoma (Duvic et al., 2001). In the cosmetic domain, retinol and other stable retinoid derivatives are incorporated into cosmeceuticals targeting signs of aging with favorable skin tolerance (Mambwe et al., 2025; Zasada and Budzisz, 2019).

**TABLE 1:** Clinical applications, dosage, and notable features of retinoids across different generations.

Retinoid type	Examples	Indication	Administration/Dosage notes	Notable features	Ref
1st Generation (natural forms)	Retinol, Retinal, Tretinoin (atRA), isotretinoin, Alitretinoin	Acne, photoaging, intrinsic aging, ichthyosis, rosacea	Tretinoin: topical 0.01%–0.1% cream/gel once daily, long-term maintenance recommended Isotretinoin: oral 0.1–1 mg/kg/day usually in two divided doses for 15–20 weeks. Strict pregnancy prevention required. Monitor lipids and liver function Alitretinoin: oral 30 mg/day for chronic hand eczema	Natural structure; effective but more irritating; isotretinoin linked to suicide risk; teratogenic	Paichitrojjana and Paichitrojjana (2023), Zasada and Budzisz (2019), Vašková et al. (2025), Reynolds et al. (2024)
2nd Generation (synthetic)	Etretinate, acitretin	Psoriasis, keratinization disorders	Etretinate: withdrawn in most countries due to long half-life Acitretin: oral 25–50 mg/day often in combination with phototherapy	Longer half-life; systemic side effects; teratogenic	Vašková et al. (2025), Uw and Rc (1998), Amanda et al. (2012), Wa et al. (2024), Joseph et al. (2008), Blanchet-Bardon et al. (1991)
3rd Generation (selective)	Adapalene, Tazarotene, Bexarotene	Acne, psoriasis, cutaneous T cell lymphoma (CTCL), photoaging	Adapalene: topical 0.1%–0.3% gel/cream daily Tazarotene: topical 0.05%–0.1% cream/gel daily, often applied at night for ≥12 weeks Bexarotene: oral 100–300 mg/m^2^/day or topical 1% gel 1–4 times daily. Often initiated at 300 mg/m^2^/day and titrated based on tolerance. Strict pregnancy prevention required. Monitor thyroid function, lipids, and liver function	Improved receptor selectivity; better tolerability	Joseph et al. (2008),; Bikash and Sarkar (2023), Baldwin et al. (2021), Guenther (2003), Gniadecki et al. (2007)
4th Generation	Trifarotene	Acne (face and trunk), photoaging	Topical 0.005% cream daily, approved for large-surface application	Highly selective for RARγ; less irritation	Baldwin et al. (2021), Roeder et al. (2004)
Cosmeceutical Derivatives	Retinol, Retinyl palmitate, Retinyl acetate	Photoaging, intrinsic aging, pigmentation	Widely used in OTC cosmetics at 0.1%–0.3% concentrations; often in long-term daily regimens	Lower potency; better stability and tolerability	Zasada and Budzisz (2019), Chien et al. (2022), Shu et al. (2024), Kajal and Reza (2010)
Specialty Retinoids	Alitretinoin (Panretin), Bexarotene (Targretin)	Kaposi’s sarcoma, CTCL	Alitretinoin: topical 0.1% gel daily Bexarotene: oral 300 mg/m^2^/day; topical 1% gel 1–4 times daily	RXR agonists; oncology use	Gniadecki et al. (2007), Kapser et al. (2015), Coors and von den Driesch (2012)
Combination Therapies	Retinoid + Hydroquinone, Retinoid + Corticosteroid, Retinoid + Erythromycin	Melasma, acne, psoriasis	Typical combinations Tretinoin (0.05%) + Hydroquinone (4%) Tretinoin (0.05%) + Corticosteroid (0.025%) Tretinoin (0.05%) + Erythromycin (3%) gel Fixed formulations such as Tri-Luma: fluocinolone 0.01% + hydroquinone 4% + tretinoin 0.05%, nightly	Enhanced efficacy; reduced irritation	McKesey et al. (2020), Reynolds et al. (2024), Susan et al. (2023), Foster et al. (1998)

This table summarizes the major classes of retinoids, their representative compounds, indications, and administration regimens. Beyond applications in acne, psoriasis, and oncology, retinoids are extensively utilized for aging and photoaging management, with topical formulations established as the gold standard for improving wrinkles, pigmentation, and dermal matrix integrity. Additionally, the table highlights advances in receptor selectivity, safety profiles, and combination regimens, which collectively enhance therapeutic efficacy while minimizing adverse effects. (Comprehensive pharmacological data can be accessed via the StatPearls database).

RA has recently attracted growing attention in regenerative dermatology. When delivered through advanced carriers such as solid lipid nanoparticles or chitosan-based hydrogels, RA exhibits improved solubility, stability, and tissue retention (Arantes et al., 2020; Oluwole et al., 2024). These delivery platforms have shown therapeutic efficacy by accelerating wound closure, reducing leukocyte infiltration, optimizing collagen deposition, and minimizing scar formation. In addition to its anti-fibrotic and immunomodulatory effects, RA also promotes pro-regenerative phenotypes in mesenchymal stem cells. Treatment of mesenchymal stem cells with all-trans RA has been shown to enhance the expression of angiogenesis- and migration-related genes, including COX-2, HIF-1, CXCR4, VEGF, and angiopoietins, thereby improving both *in vitro* cell behavior and *in vivo* wound healing outcomes (Pourjafar et al., 2017). The regenerative potential of RA further extends to the reconstitution of skin appendages. Recent studies have shown that RA can induce the differentiation of human induced pluripotent stem cells (iPSCs) into dermal papilla-like cells (DPCs) (Lv et al., 2024), which are essential for hair follicle formation (Ji et al., 2021). RA-induced pluripotent stem cell-derived multipotent mesenchymal cells, when transplanted in combination with keratinocytes, are capable of forming hair follicle-like structures *in vivo* (Veraitch et al., 2017). Notably, in murine skin wound, topical application of RA has also been shown to induce hair follicle neogenesis (Tierney et al., 2024). Sustained RA treatment in a study by the Fuchs’ group successfully reactivated follicular neogenesis at wound sites, offering proof-of-concept for RA-based strategies in functional skin regeneration (Tierney et al., 2024).

Together, these findings support the integration of RA into next-generation regenerative therapies. By simultaneously modulating fibrosis, stem cell behavior, vascularization, and appendage formation, RA represents a uniquely versatile molecule for promoting comprehensive skin repair beyond mere scar attenuation.

## 5 Conclusion and future perspective

Retinoids represent a unique class of compounds that bridge dermatologic therapy and regenerative medicine. Their well-established efficacy in treating acne, psoriasis, photoaging, and selected malignancies is now being complemented by emerging roles in scar modulation and appendage regeneration. Acting through nuclear RARs, RA regulates a diverse set of biological processes, including keratinocyte differentiation, fibroblast activation, ECM remodeling, and immune modulation, which are core elements of both wound healing and fibrosis. Mechanistic studies have identified RA as a key regulator of myofibroblast differentiation and collagen deposition, largely through inhibition of TGF-β signaling. Additionally, RA enhances the regenerative capacity of stem cells and supports the restoration of skin appendages such as hair follicles. These properties collectively position RA as a potential driver of adult scarless wound healing, a long-sought goal in regenerative dermatology.

Despite promising therapeutic potential, the clinical translation of RA-based regenerative strategies faces challenges derived from poor aqueous solubility, light and oxidative sensitivity, and systemic toxicity at pharmacologic doses (Ferreira et al., 2020; Nau, 2001; Collins and Mao, 1999). Although topical use minimizes systemic absorption, the risk remains for high-potency compounds or poorly controlled formulations. These issues may be addressed by future advances in receptor-selective ligand design, smart delivery systems, and controlled release technologies. The integration of RA into combination therapies, such as stem cell transplantation, tissue-engineered scaffolds and gene-modulated regenerative systems, could hopefully enhance healing outcomes. Elucidating the temporal and spatial dynamics of RA signaling during wound repair would be essential for maximizing therapeutic benefit while minimizing risk. With continued progress, retinoids may transition from symptomatic treatment agents to molecular modulators capable of promoting comprehensive skin regeneration.
